# A Rare Case of Gastrointestinal Tract Foreign Body; Glassy Stomach

**Published:** 2017-01-14

**Authors:** Mohsen Ebrahimi, Jafar Malmir, Azadeh Mahmoudi-Gharaee, Mahdi Foroughian

**Affiliations:** 1 Department of Emergency Medicine, Imam Reza Hospital, Mashhad University of Medical sciences, Mashhad, Iran.; 2 Department of Emergency Medicine, Afzalipour Hospital, Kerman University of Medical sciences, Kerman, Iran.

**Keywords:** Foreign bodies, glass, gastrointestinal tract, emergencies, case management

## Abstract

Ingestion of foreign bodies is common and conservative treatment can eliminated most particlesfrom the gastrointestinal tractunless peritoneal signs appear. A 22-year-old man presented to emergency department who had ingested glass particles of a crushed beveragebottle.Hecomplained ofepigastric and periumbilical pain. Physical examination did not revealany peritoneal signs. Abdominal X-ray showed stomach full of small glass particles. Conservative treatment, without any surgical intervention,resulted insafely eliminating glass particlesin this patient.

## Introduction

Foreign bodies may be ingested, inserted into a body cavity, or deposited into the body by a traumatic or iatrogenic injury. Most ingested foreign bodies pass throughthe gastrointestinal tract without any problem(1). However, ingested or inserted foreign bodies may cause bowel obstruction or perforation and lead to serious complications such as severe hemorrhage, abscess formation, or septicemia.Most cases of foreign body ingestions are seen amongchildren and those with psychiatric disorders(2-4). Here we report a case of intentional foreign body ingestion and itsoutcome.

## Case presentation

A 22-year-old male was brought to emergency department with chief complaint of periumbilical and epigastric pain since 4 hours before. He explained that he had ingested crushed glasses of a beverage bottleafter a family argument. About 30 minutes after swallowing glass particles,the patient had developed irritation and pain in epigastric and periumbilical area without any other associated symptom.Hedenied history of other diseases including psychiatric problems.On physical examination normal vital signs were detected, no oral lesion or laceration or bleeding was seen. He had normal breathing sounds. Abdomen had normal bowel sounds with mild epigastric tenderness without rebound tenderness or guarding.

**Figure 1:**
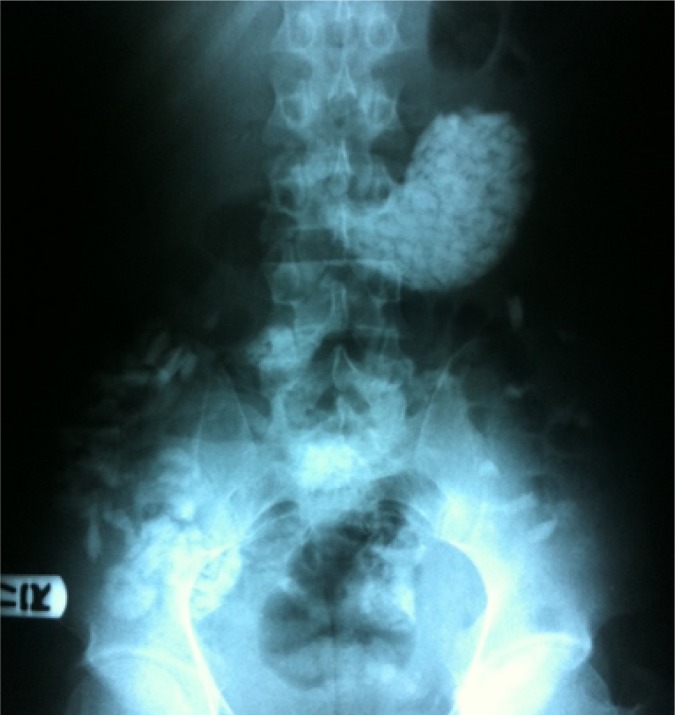
Upright abdominal X-ray showing a stomachfull of glass particles

**Figure 2:**
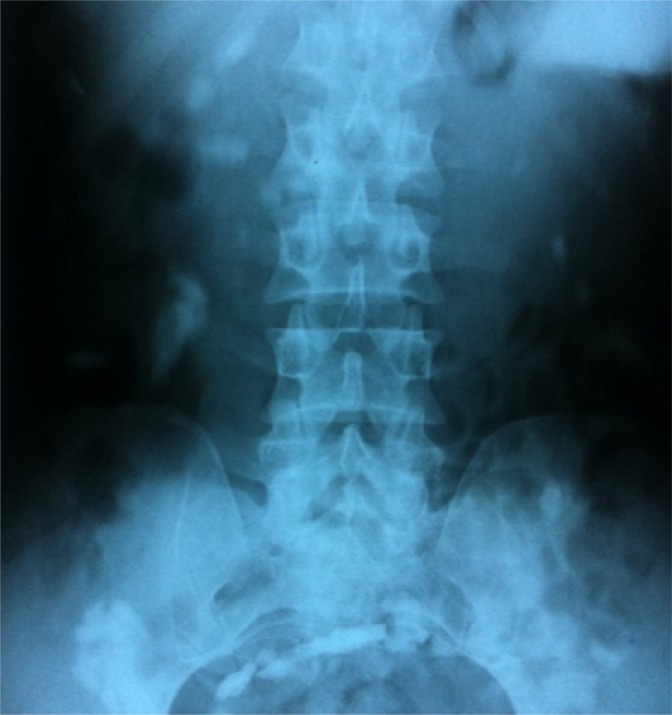
Upright abdominal X ray, 48 hours after glass ingestion (the glass particles are in the large intestine).

Chest X-ray and abdominal X-ray were performed. Chest X-ray was normal with no air under diaphragm or pneumomediastinum. Abdominal X ray (figure 1) showed a stomach full of small glass particles.

The patient was monitored continuously and carefully; surgical consultation was done, laparotomy was planned but patient didnot agree to undergo surgical intervention.Thereafter, he was monitored closely and observed in emergency department with serial physical exam. Serial abdominal radiography was done every other day to evaluate the removal of the particles (figure 2). All glass particles were eliminated from the gastrointestinal tract of patient without any intervention.No complication was seen during the observation period. Eventually after 10 days the patient was discharged and went home.

## Discussion

Cases of intentional glass ingestionare rare, so there is no special guideline to approach them. In these cases, it is expected to see oral cavity laceration, drooling, inability to swallow, neck pain or chest pain. If the objects could pass the esophagus, mild abdominal pain or even signs of acute abdomen may appear. Based on the routine approach, in order to identify the location, number and size of the ingested particles, and also evaluate the presence of any kind of complications, radiography could be suggested as an initial screening method(5). Although glass foreign bodies are opaque on radiographs, but it was indicated that the size of the glass foreign body is often the limiting factor for radiographic detection and that 0.5- to 2.0-mm fragments represent a “limited detection” size range(1, 6).Endoscopic extraction is well accepted and recommended as a form of treatment for swallowed foreign body in upper gastrointestinal tract, however, conservative approach with proper management is also effective and preferable when foreign bodies have passed the esophagus within days without any difficulty (7).This is the treatment of choice for blunt, short (<6 cm), and narrow (<2.5 cm diameter) foreign bodies, especially once they have passed the pylorus(8). 

Emergency esophagogastroduodenoscopy is suggestedin cases of sharp or pointed foreign bodies. They can result in complications such as gastrointestinal bleeding, abscess formation,mediastinitis or peritonitis due to perforation of the gastrointestinal tract(9, 10). Surgical intervention is required in such cases, which make up less than 1% of ingested foreign body cases(11). 
